# Implementation and analysis of initial trauma registry in Iquitos, Peru

**DOI:** 10.15171/hpp.2016.28

**Published:** 2016-10-01

**Authors:** Vincent Duron, Daniel DeUgarte, David Bliss, Ernesto Salazar, Martin Casapia, Henri Ford, Jeffrey Upperman

**Affiliations:** ^1^Department of Pediatric Surgery, New York-Presbyterian Morgan Stanley Children’s Hospital, Columbia University Medical Center, New York, USA; ^2^Department of Pediatric Surgery, Mattel’s Children Hospital, University of California, Los Angeles, CA, USA; ^3^Department of Pediatric Surgery, Children’s Hospital Los Angeles, University of Southern California, Los Angeles, CA, USA; ^4^Chief of Surgery, Department of Surgery, Hospital Regional Loreto, Punchana, Iquitos, Peru; ^5^Chief of Medicine, Department of Medicine, Hospital Regional Loreto, Punchana, Iquitos, Peru

**Keywords:** Global health, Injury, Trauma Registry

## Abstract

**Background:** In Peru, 11% of deaths are due to trauma. Iquitos is a large underserved Peruvian city isolated from central resources by its geography. Our objective was to implement a locally driven trauma registry to sustainably improve trauma healthcare in this region.

**Methods:** All trauma patients presenting to the main regional referral hospital were included in the trauma registry. A pilot study retrospectively analyzed data from the first two months after implementation.

**Results:** From March to April 2013, 572 trauma patients were entered into the database. Average age was 26.9 years. Ten percent of patients presented more than 24 hours after injury. Most common mechanisms of injury were falls (25.5%), motor vehicle collisions (23.3%), and blunt assault (10.5%). Interim analysis revealed that 99% of patients were entered into the database. However, documentation of vital signs was poor: 42% of patients had temperature, 26% had oxygen saturation documented. After reporting to registry staff, a significant increase in temperature (42 to 97%, P < 0.001) and oxygen saturation (26 to 92%, P < 0.001) documentation was observed.

**Conclusion:** A trauma registry is possible to implement in a resource-poor setting. Future efforts will focus on analysis of data to enhance prevention and treatment of injuries in Iquitos.

## Introduction

The global burden of surgical disease has recently been recognized as one of the foremost contributors to worldwide mortality and loss of disability-adjusted life years (DALYs).^1,2^ In fact, 16% of the global burden of disease can be treated with surgery.^3^ Most of the deaths from surgical disease are attributable to injury and trauma, which is even more pronounced in developing countries.^1^ While trauma is responsible for 5%-6% of deaths in industrialized countries, it causes at least 10%-13% of deaths in developing countries.^4^ In Peru, 10.7% of deaths are due to trauma.^5^ Injuries also cause 15% of the world’s DALYs – a significant contributor to individual, family, and community losses in productivity and quality of life. South America is third in the world in terms of DALYs lost from injury and trauma.^6^

Iquitos is the largest city in the Peruvian rainforest and the capital of the Loreto region and Maynas province in the North of Peru. The city is considered the sixth largest city of Peru, with 457 865 inhabitants.^7^ However, it is geographically very isolated, attainable only by air or water. Fifty-five percent of the population is considered to be living under the poverty line, and 20% live in extreme poverty.^8^ Amazonians have among the lowest educational level throughout Latin America due to a failed education structure and lack of opportunity.^9^ Access to healthcare resources is also severely lacking, although limited data has been published in the international press on health indices in Iquitos. Our objective was to implement a locally driven health intervention through disease stakeholder engagement and disease identification. We designed this project to be a locally supported and developed health promotion project to fill a self- identified knowledge gap and improve healthcare in the Loreto region.

## Materials and Methods

### Participants and procedures

In March 2013, a needs assessment was completed to determine the surgical burden of disease in Iquitos and which intervention would be beneficial to improving surgical care in the region. The project site was the Hospital Regional Loreto (HRL), the main referral hospital of the region – 249 beds, 44 ICU beds. As trauma is the foremost contributor to mortality and DALYs, efforts were focused on improving care of injured individuals. It was determined that no trauma registry exists for HRL or any surrounding hospital. Trauma registries play an integral role in the assessment of trauma care quality and allow the improvement in hospital based trauma care through an evidence-based analysis of prospectively collected data.

We hypothesize that the implementation of a trauma registry is feasible with existing local resources, and that it will sustainably improve trauma healthcare in this underserved region of Peru. The first step of this project was the implementation of the trauma registry. A pilot analysis of the data gathered through the trauma registry over the first 2 months was then performed to determine the accuracy and sustainability of this database.

Based on the results of the initial needs assessment, a trauma registry was created in March 2013. The results from the focus groups (Table 1) and analysis of hospital epidemiological data sheets guided the creation and implementation of this context-specific trauma registry for the HRL. We incorporated elements from prior successful trauma registries and trauma guidelines established by the World Health Organization (WHO).^10-12^ All trauma patients of all ages (pediatric and adult) as characterized by ICD-9 E800-999 codes who presented to HRL were included in the trauma registry. The registry was to be comprehensive but also straightforward to complete. It was to fit on one sheet of paper to facilitate charting. The need to fill out “other” categories was minimized to facilitate future data analysis. Efforts were made to include data points that were clinically useful. The Revised Trauma Score, Glasgow Coma Scale, and Lund Browder burn diagram were included for quick reference.

Multiple editions of the registry were completed until the HRL trauma registry was appropriately adapted to the local context. Animal bites, for example are very common in Iquitos, in particular dog and rat bites during winter months. This injury mechanism was thus added and sub-classified in the registry. The presence of alcohol or drug use could only be detected subjectively by the initial responder’s examination of the patient’s demeanor, behavior and breath. This category was eliminated from the registry due to lack of objective measurement. Oil burns are very common in the region. These were added to the registry; chemical burns on the other hand are rare and so were removed from the registry. Finally, the flow of the trauma registry sheets (or “trauma sheets”) was altered to better mimic the trajectory of the patient throughout his/her hospital stay. Each patient entry had 180 possible data points. The trauma sheet was written in the local Spanish language (Supplementary file 1 – English translation).

### Measures

**Figure 1:**
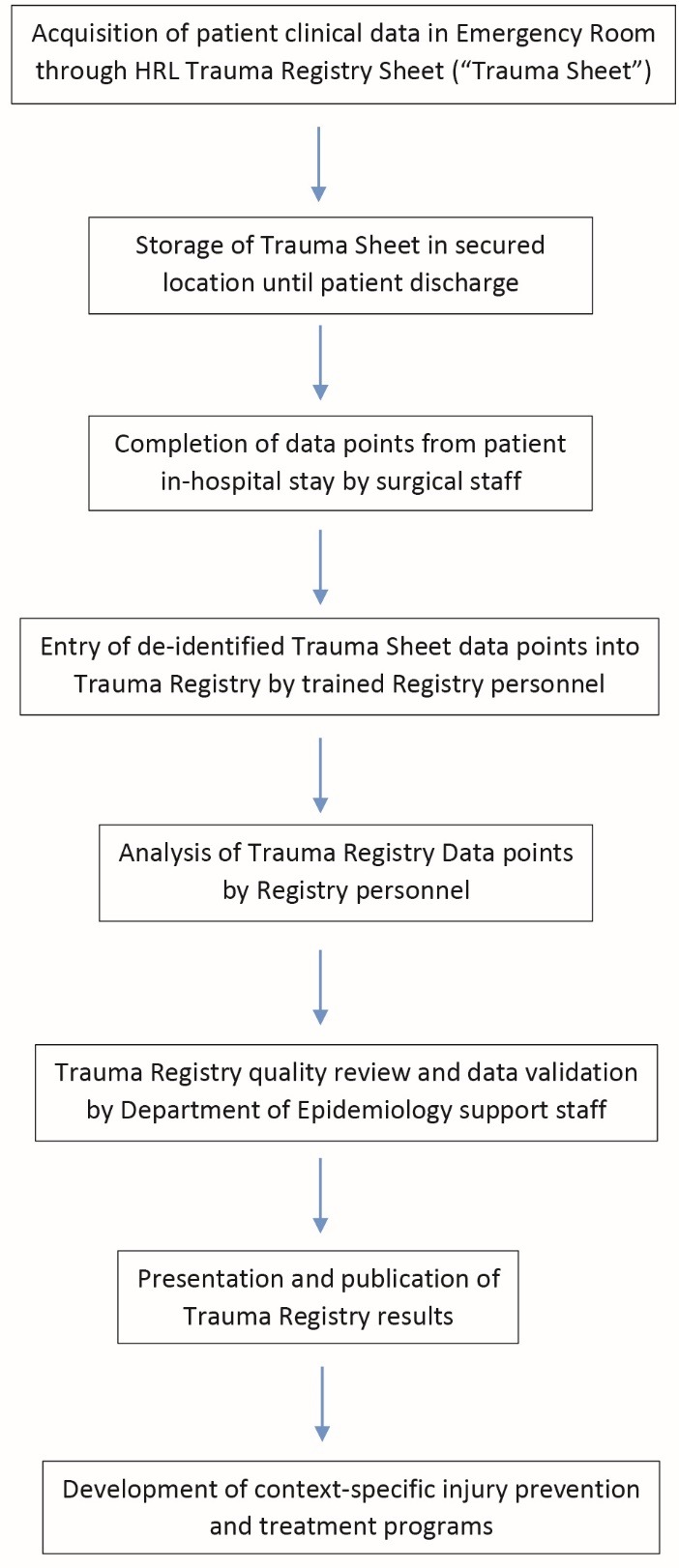
Hospital Regional Loreto Trauma Registry data flowchart.

The HRL Trauma Registry was then implemented on March 7, 2013. The paper trauma sheet was completed by emergency department (ED) trauma first responders – surgical interns rotating in the ED. To prevent misplacement or loss, the trauma sheets were not included in the paper chart, which itself is frequently misplaced. All trauma sheets were placed in a labeled envelope and delivered to the Chief of Surgery administrative assistant on a daily basis. It was kept under lock and key in the Chief of Surgery’s office, which was itself locked. Protection of patient health information was ensured in the trauma sheets as would be for any element of the patient’s chart. Once the trauma sheet was no longer being used for patient care, it was de-identified with no patient identifiers remaining. The surgery administrative assistant was well trained in data entry and thus was chosen to enter the trauma sheet data points into the HRL Trauma Registry, which was in excel format. This assignment of tasks was found by the Chief of Surgery to be the most likely to avoid incomplete registries and mistakes. A flowchart of the Trauma Registry is included (Figure 1).

The trauma data entry was overseen by the study authors and an interim analysis was performed after 10 days to validate the registry and improve accuracy. Subsequently, a pilot study retrospectively analyzed data from the first two months after implementation of the registry, from March 7, 2013 to May 12, 2013.

### Statistical Analyses

Results were obtained through quantitative analysis of the data using sum and mean calculations. Student *t* tests were used to compare continuous data and Fisher exact tests were used to compare categorical data before and after the 10-day intervention, with α = 0.05. All statistical analyses were performed using Stata version 14 software (StataCorp. 2015. Stata Statistical Software: Release 14. College Station, TX: StataCorp LP).

## Results

Over 2 months, 572 patients presented to HRL for trauma. The average age of trauma patients was 26.9 years (SD 20.8, min 0, max 101). Sixty-five percent were males (n = 374). Pediatric patients (18 years and below) made up 38.5% of patients (n = 220). Of pediatric patients, 64% (n = 140) were boys. Fewer than 2% (1.9%, n = 11) of patients were less than 1 year of age. Most patients (75%) presented within 3 hours of injury. However a considerable number of patients (9.6%) presented more than 24 hours after trauma. Most patients presented to the hospital by mototaxi (71%) – a three-wheeled motorcycle-rigged taxi. Only 2.2% arrived by ambulance (Table 2). Overall, the most common mechanisms of injury were falls (25.5%), motor vehicle collisions (23.3%), blunt assault (10.5%), stab wounds – intentional and accidental (7.8%), and animal bites (7.6%). Among pediatric patients, the most common cause of injury were motor vehicle collisions (23.1%) (Table 3). Motorcycles were most frequently involved in motor vehicle collisions in both adults and children (Table 4).

The most common ED procedures were for wound management - 30% of patients had a laceration repair and 48% had a wound washout in the ED. Although the indications were infrequently reported, 7% of patients received antibiotics in the ED. Two percent of patients had a procedure performed in the OR (Table 5). Of all patients, 83% were sent home directly from the ED, 3% were transferred to another facility, and 12% of patients were admitted to the inpatient ward. Among patients admitted to the hospital, two thirds were admitted to the surgical service and a quarter of the patients were admitted to the ICU.

After 10 days of data entry, an interim analysis was undertaken to ensure internal validity and improve accuracy. At this point, data on 145 patients had been entered into the registry. Analysis of the gathered data points and comparison to the ED logbook revealed that 99% (143/145) of patients presenting to HRL with trauma had been entered into the database. However, it was also noted and reported to registry staff that a number of sections were incompletely documented. Entry of vital signs was poor: only 42% of patients had temperature documented and 26% had oxygen saturation documented on arrival to ED. Following this intervention, there was a statistically significant increase in temperature documentation (42% to 97%, *P* < 0.001) and oxygen saturation (26% to 92%, *P* < 0.001) documentation.

Interestingly, documentation of the date of discharge and length of hospital stay was worse in the later period of data entry (Table 6). The research coordinator (study author) was on site for the first month of data collection. Part of his responsibility was to ensure completion of all trauma registry sheets throughout the patient’s hospital stay. It is likely that his departure contributed to decreased patient follow up.

**Table 1:** Focus groups

1. Hospital Regional Loreto (HRL) Directors – Medical Director HRL, Chief of Surgery, ex-director of HRL as of Feb 2013
2. Department of Surgery Staff – 4 general surgery attending, chief of surgery
3. Department of Pediatrics Staff – chief of department of pediatrics, pediatrics residents (2), PICU attending (1), NICU attending (1)
4. Anesthesia and Surgery Staff – Chief of anesthesia, anesthesia attending (2), surgery attending (2)
5. Personal Interviews (5) – local pediatric surgeon, chief of surgery, ex-director HRL, chief of second largest regional hospital, Hospital Iquitos

**Table 2:** Patient demographics and pre-hospital data

	**Number**	**Percent**
Demographics		
Gender – Male	374	65.4
Female	198	34.6
Pediatric patients	220	38.5
Less than 1 year old	11	1.9
Pre-hospital		
Time to Presentation		
Within 3 hours of injury	429	75
Within 3-12 hours	60	10.5
Within 12-24 hours	26	4.6
More than 24 hours after injury	55	9.6
Mode of Transport		
Moto taxi	406	71
By foot	69	12
Ambulance	13	2.2
Boat	8	1.4
Car	4	0.7

**Table 3:** Mechanism of injury

**Overall (n = 572)**	**Pediatric (n = 220)**
Fall: 25.5% (n = 158)	Motor vehicle collision: 23.1% (n = 51)
Motor vehicle collision: 23.3% (n = 144)	Fall: 20.8% (n = 46)
Blunt assault: 10.5% (n = 65)	Stab wound: 13.6% (n = 30)
Stab wound: 7.8% (n = 48)	Blunt assault: 12.7% (n = 28)
Animal bite: 7.6% (n = 47)	Animal bite: 9.5% (n = 21)
Gun shot wound: 0.6% (n = 4)	Gun shot wound: 0.5% (n = 1)

**Table 4:** Motor vehicle collisions

**Overall: (n = 144)**	**Pediatric: (n = 51)**
Auto: 3.4% (n ?= ?5)	Auto: 7.8% (n = 4)
Moto taxi: 20.6% (n = 30)	Moto taxi: 10% (n = 5)
Motorcycle: 43.8% (n = 63)	Motorcycle: 27.3% (n = 14)
Bicycle: 0% (n = 0)	Bicycle: 0% (n = 0)
Pedestrian vs. auto: 2.6% (n = 4)	Pedestrian vs. auto: 7.8% (n = 4)
Pedestrian vs. Moto taxi: 13.7% (n = 20)	Pedestrian vs. Moto taxi: 23.4% (n = 12)
Pedestrian vs. motorcycle: 14.6% (n = 21)	Pedestrian vs. motorcycle: 21.5% (n = 11)

**Table 5:** Surgical interventions

**Intervention**	**Number** ^a^	**%**
Hemi craniotomy	1	0.2
Exploratory laparotomy with bowel resection	1	0.2
Digital amputation	2	0.4
Complex wound closure	2	0.4
Chest tube	2	0.4
Fasciotomy	1	0.2
Orthopedic procedure	3	0.5

^a^Each patient had only one procedure performed in the OR.

**Table 6:** Interim analysis

**Parameter**	**Percent recorded: Pre-intervention** **(n = 142)**	**Percent recorded: Post-intervention** **(n = 430)**	***P*** **value** ^a^
Age	98% (n = 139)	100% (n = 430)	0.015
Length of stay	96% (n = 137)	90% (n = 391)	0.030
Vitals on admission			
Temperature	42% (n = 61)	97% (n = 418)	<0.001
Pulse	98% (n = 139)	99% (n = 429)	0.049
SBP/DBP^b^	98% (n = 140)	99% (n = 427)	0.602
SaO2^b^	26% (n = 37)	92% (n = 394)	<0.001

Abbreviations: SBP/DBP, systolic blood pressure/diastolic blood pressure; SaO2, arterial oxygen saturation. ^a^Fisher’s exact tests were use to calculate statistical significance pre/post intervention

## Discussion

Through the focus groups performed and the pilot study completed, we are confident that a valid trauma registry can be implemented in the HRL to sustainably improve the health of the Iquitos community. To make our project a successful global health initiative, we wanted to shift away from short-term unsustainable interventions such as trainee exchange, equipment donation, and clinical service delivery models such as short mission trips.^13^ We focused on long-term capacity building, the aims of which were determined by local healthcare providers as it has been suggested that locally driven and sustained healthcare projects are the most likely to succeed.^14,15^

Injury surveillance is a key element in developing resource and culture specific interventions to enhance prevention and treatment of injuries.^16^ Furthermore, such evidence-based capacity building may attract local and outside funding to aid in hiring new personnel, implementing community based injury prevention initiatives, and providing contextually specific training of healthcare providers.^17^ For these reasons, and with the guidance of local healthcare leaders through focus groups and interviews, the establishment of a trauma registry was undertaken.

Multiple challenges exist in creating a valid trauma registry in an underserved setting. The lack of programmed trauma documentation was both the reason for but also the main impediment toward ensuring the accurate documentation of clinical data. The clinicians at HRL are not accustomed to using a pre-established itemized note when documenting their patient encounters. The first step was pulling them away from patient focused documentation, in which they record only pertinent findings, and shifting to a standardized mode of documentation. The initial documentation of trauma patients is completed by surgical interns, who are simultaneously very motivated but also variable in their skill set. The chief of the surgery department was intimately involved in the creation of this registry, which was crucial to ensuring reliable data documentation. We concluded that the manpower for data entry is available – in the form of educated surgical interns and dedicated senior attending staff. The Hospital’s Epidemiology Department has also committed to assisting in quality review and data validation. The time spent per case is not overwhelming and does not overburden the surgical trainees. In fact, it educates them in proper medical documentation and focused patient evaluations. There was no cost to recruitment, as this was part of the medical evaluation and chart. A challenge of the program is providing continuity in training the staff evaluating the patients and entering the data.

This study has a number of limitations, including potential reporting bias and recall bias. In addition, many of our conclusions are inferred rather than statistically demonstrated. However, we feel that we were able to obtain valid data points from our pilot study. The fact that almost all (99%) of the patients who presented to HRL were entered into the trauma registry was the initial step in validating the feasibility of the registry. The lack of reliable duplicated documentation, such as initial History and Physical or Consult notes, makes it impossible to confirm the accuracy of the documentation however, which is a limitation of the study.

The interim analysis performed after 10 days of data-entry identified weaknesses in vital signs documentation, specifically oxygen saturation and temperature. Once identified, these deficiencies were quickly rectified and there was a significant improvement in documentation. Interestingly, there was a significant decrease in the number of patients who had complete documentation of length of stay. As previously mentioned, the research coordinator (study author) was on site for the first month of data collection, and ensured completion of all trauma registries. We deduced that his departure most likely contributed to this decline in the quality of documentation. This finding highlights the importance of a dedicated on-site trauma registry coordinator.

In terms of potential interventions, several findings hold promise for the usefulness of the trauma registry in improving care. It was documented that a significant percentage (10%) of patients had a delayed presentation after trauma (>24 hours). Research into the barriers in access to care that exist in Iquitos would be beneficial. Motorcycles were most frequently involved in motor vehicle collisions. Analysis of injury pattern and patient population could help focus intervention strategies. Almost half of patients (48%) received wound management in the ED, yet supplies are scant in this department and instead are scattered throughout the hospital, sometimes remaining unused past expiration dates. Also, most patients (83%) were discharged directly from the ED. Mobilization of supplies to the ED should occur, and focus on disposition and follow up appointments should take place in the ED.

Finally, the indication of antibiotic use was rarely documented, and only 7% of patients were documented as having received antibiotics. Yet, when one considers that already 7.6% of patients presented with animal bites, which almost always warrant antibiotics, it seems that the percentage of patients receiving antibiotics may be too low or incompletely documented. Prior to inferring that antibiotics are not prescribed appropriately, specific attention needs to be placed on confirming that antibiotic use is being accurately documented in the registry.

This pilot study has highlighted the feasibility of establishing and administering a trauma registry based out of the HRL – a large referral hospital in a resource poor area. It has also suggested the need for dedicated staff and constant supervision of data entry to guarantee validity. The next step in the project is to ensure continuous funding for this purpose. The HRL is the main teaching hospital for the National University of the Peruvian Amazon Medical School (Escuela de Medicina de la Universidad Nacional de la Amazonia Peruana) - a 40-student medical school that aims to train medical doctors in the hopes that they will serve their local community. We hope to contribute to the local care of trauma patients by offering healthcare providers with sound, prospectively collected data on which they may base their decision-making. Finally, an overarching goal of this project is to expand the trauma registry to other regions of Peru and ultimately create a national trauma registry, which does not yet exist in this country.

Three years have passed since the results of this study were obtained. The registry has been intermittently maintained and analyzed. The challenges in continuously training staff for data entry have proven significant. We have discovered deficiencies in the training that was passed on after the initial phase of the study, further demonstrating the need for a permanent on site trauma coordinator. Over this time, we have obtained funding for a trauma coordinator. The trauma coordinator will be recruited to re-initiate the trauma registry this August 2016. The inclusion of an on-site trauma coordinator will allow maintenance and sustainability of the trauma registry. We have the support of the local Ministry of Health and the support of all levels of administration in the HRL, which makes the Trauma Registry a locally authenticated program. We hope that recent developments in funding and staff management will make it a long term sustainable program.

## Implication for policy and practice

The lack of continuity in the training of the caregivers entering the data has been the primary challenge we have been faced with. This echoes findings from other studies which have found it to be the most important aspect of maintaining a registry – training an on-site trauma coordinator who oversees all registry data entry.^18,19^ This allows validity of the data and in-hospital follow up to include all data points until patient discharge. With support from the Ministry of Health and various funding sources, a trauma coordinator will start working this year.

We hope that this registry will lead to new trauma practice guidelines allowing the standardization of trauma care. More studies focusing on future specific intervention strategies are needed. We plan on working with the local branch of the Ministry of Health and Department of Epidemiology to initiate these studies that we hope will lead to policy changes focusing primarily on injury prevention.

In conclusion, this pilot study is the first step to standardizing trauma care in the Loreto region. It will allow locally-driven improvement in prevention and treatment of injured individuals.

## Acknowledgments

The authors would like to acknowledge the Hospital Regional de Loreto Department of Surgery and the Department of the Chief Medical Officer for their logistical assistance.

## Ethical approval

The study was granted approval from the ethical review boards of the principal investigator’s institution – Children’s Hospital Los Angeles (IRB CCI-13-00073 and CCI-13-00120). The study also had full approval of the Medical and Surgical Board of HRL in Iquitos, Peru. Board approval was obtained twice – initially for the needs assessment portion of the project and subsequently for the trauma registry data analysis portion.

## Competing interests

There are no competing interests.

## Authors’ contributions

VD contributed to project design, data collection, analysis, manuscript drafting and editing. ES and MC contributed to project design, data collection, and data analysis. DD, DB, HF and JU contributed to project design, and manuscript editing.

## Supplementary materials

Supplementary file 1 contains the HRL trauma registry form.
