# Supplementary material for: Implementation and analysis of initial trauma registry in Iquitos, Peru
Source: Health Promot Perspect. 2016 Oct 1;6(4):174–9. doi: 10.15171/hpp.2016.28 (PMC5071783; doi:10.15171/hpp.2016.28)
Supplement: Supplementary file 1 [file hpp-6-174-s01.pdf]

DPTO. CIRUGÍA

HOSPITAL REGIONAL DE LORETO "Felipe Arriola Iglesias" Te Sirve con ....

Trauma Sheet

1.__________________________ Last Name

2.__________________________ First Name

3.____________________ Medical Record Number

4. _____/_____/_____ Date of Birth

5. __________ Age

6. Male____ Female____ Gender

7. ______________________________ District

8. ______/______/______ Date of admission

9. ____________ Time of arrival

10. ___________________ Mode of transport

11. ______________________ Transferred from

12. Vital Signs: Temp: ____ Pulse: ____ BP: ___/___ RR:____ Sat O2: ____ Glasgow: E___ V___M___ Total: ___

13. Revised Trauma Score: Glasgow: ___/4 RR:___/4 BP: ___/4 TOTAL: _____/12

14. Time since trauma: □ <3hrs □ <12hrs □ <24hrs □>24hrs

15. Mechanism of Injury:

□ Vehicle: □ Car □ Mototaxi □ Motorcycle □ Bicycle □ Other___________ □ Animal Bite: _______

□ Pedestrian/□ Bicycle against: □ Car □ Mototaxi □ Motorcycle □ Other___________

□ Fall: from: _____ height: ___ meters □ Stab wound □ Gun shot wound □ Assault □ Other mechanism: ____________

□ Burn: □ Water □ Oil □ Flame □ Gasoline □ Explosive □ Electric (voltage:______) □ Other: ______________________

Percentage: 1st____% 2nd____% 3rd____% 4th ____% Total: ______%

16. Area of Body Injured:

□ **Cranial:** □ Laceration □ Contusion □ Fracture: □ Closed □ Open □ Contaminated

□ TBI: □ Light (GCS 14-15) □ Moderate (GCS 9-13) □ Severe (GCS 3-8)

□ ***Face***: □ Laceration □ Contusion □ Fracture □ Dislocation □ Eye injury □ Dental injury

□ ***Neck***: □ Penetrating □ Blunt □ Carotid Injury □ Tracheal Injury □ Laryngeal Injury

□ ***Spine***: □ Ligament □ Dislocation □ Fracture: □ Stable □ Unstable □ Cervical □ Thoracic □ Lumbar □ Sacral

□ ***Thorax***: □ Penetrating □ Blunt □ Fracture: □ Clavicle □ Sternum □ Rib: # of ribs ___________

□ Pneumothorax □ Hemothorax □ Pulmonary Contusion □ Cardiac Injury: □ Penetrating □ Blunt

□ ***Abdominal***: □ Penetrating □ Blunt □ Spleen □ Liver □ Intestinal □ Bladder □ Kidney

□ ***Pelvis***: □ Laceration □ Contusion □ Fracture: □ Open □ Closed □ Stable □ Unstable □ Contaminated

□ ***Urogenital:*** □ Penetrating □ Blunt □ Penile □ Scrotal □ Vaginal □ Urethral □ Rectal

□ ***Upper Extremity***

□ *Left*: □ Laceration □ Contusion □ Dislocation

□ Fracture: □ Open □ Closed □ Contaminated □ Humerus □ Forearm □ Hand

□ *Right*: □ Laceration □ Contusion □ Dislocation

□ Fracture: □ Open □ Closed □ Contaminated □ Humerus □ Forearm □ Hand

□ ***Lower Extremity***

□ *Left*: □ Laceration □ Contusion □ Dislocation

□ Fracture: □ Open □ Closed □ Contaminated □ Femur □ Patellar □ Tibia/Fibula □ Ankle □ Foot

□ *Right*: □ Laceration □ Contusion □ Dislocation

□ Fracture: □ Open □ Closed □ Contaminated □ Femur □ Patellar □ Tibia/Fibula □ Ankle □ Foot

□ ***Other***: ____

17. Diagnostic Imaging: □ X-Ray □ Ultrasound □ Tomography

Findings: ____

18. Surgery (in OR): ____

19. Interventions (in Emergency Room): □ Laceration Repair □ Washout □ Dressing in ER □ Dressing in OR

□ Splint □ Casting □ Traction □ Amputation □ Chest tube □ Thoracotomy □ Antibiotics

□ Other: ____

20. Hospitalization: Admission Floor: □ ICU □ Surgery □ Medicine □ Pediatrics □ Observation

□ Discharged from ER □ Transferred to __________________

21.____________ Days in ICU

22.________________ Days on ventilator

23._____ /_____ /______ Discharge date from hospital

24. **Disposition**: □ alive □ dead

Signature of Provider: ____ **Date:** ______/_____/_____

Revised Trauma Score:

Is made up of 3 categories: Glasgow Coma Scale, blood pressure (systolic), and respiratory rate. The score ranges from 0-12. The lower the score, the more severe the trauma. A patient with the following point total requires the following attention:
12 – attention without urgency; 11 – urgent attention; 3-10 – immediate attention; <3 – little chance of survival
The score is calculated as follows: Glasgow Coma Scale + Systolic Blood Pressure + Respiratory Rate = TOTAL

GLASGOW COMA SCALE

GCS	Points
15-13	4
12-9	3
8-6	2
5-4	1
3	0

SYSTOLIC BLOOD PRESSURE

SBP	Points
>89	4
76-89	3
50-75	2
1-49	1
0	0

RESPIRATORY RATE

RR	Points
10-29	4
>29	3
6-9	2
1-5	1
0	0

Burns:

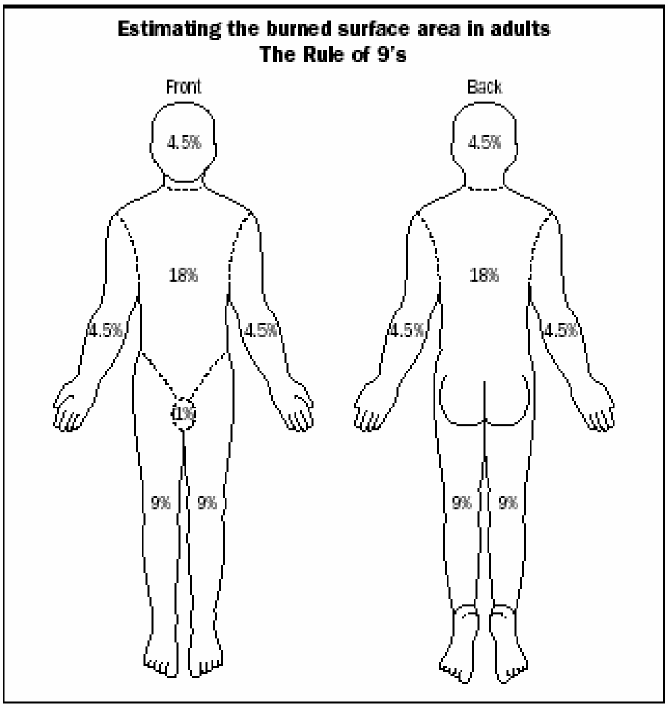

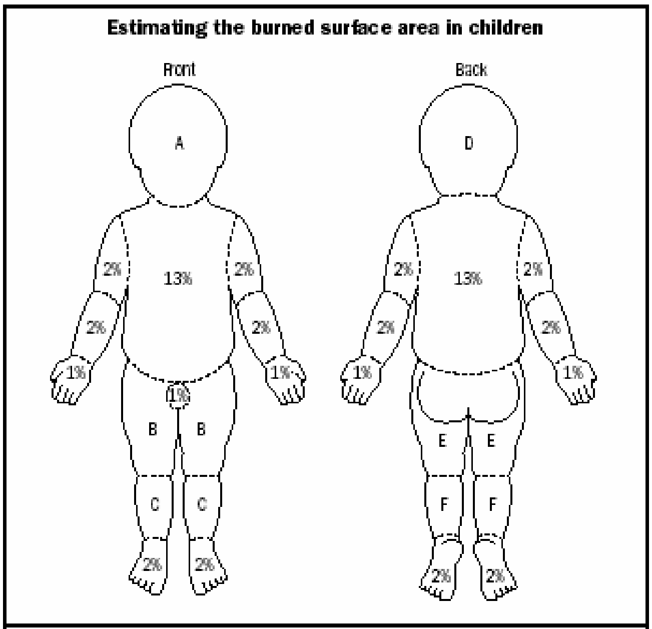

Area	By age in years			
	0	1	5	10
Head (A/D)	10%	9%	7%	6%
Thigh (B/E)	3%	3%	4%	5%
Leg (C/F)	2%	3%	3%	3%

Glasgow Coma Scale		
Eye Opening (E)	Spontaneous	□ 4
	To Voice	□ 3
	To Pain	□ 2
	None	□ 1
Verbal Response (V)	Oriented	□ 5
	Confused	□ 4
	Inappropriate Words	□ 3
	Incomprehensible Words	□ 2
	None	□ 1
Motor Response (M)	Follows Commands	□ 6
	Localizes Pain	□ 5
	Withdraws from Pain	□ 4
	Flexion to Pain	□ 3
	Extension to Pain	□ 2
	None	□ 1
	TOTAL :	

References:

Schultz CR, Ford HR, Cassidy LD, Shultz BL, Blanc C, King-Schultz LW, Perry HB. Development of a Hospital-Based Trauma Registry in Haiti: An Approach for Improving Injury Surveillance in Developing and Resource-Poor Settings. *J Trauma.* 2007;63:1143–1154.
Mock C, Lormand JD, Goosen J, Joshipura M, Peden M. Guidelines for essential trauma care. Geneva, World Health Organization, 2004.
WHO Integrated Management Package on Emergency and Essential Surgical Care (IMPEESC); WHO manual Surgical Care at the District Hospital (SCDH). Geneva, World Health Organization, 2003.
